# Role of Perineal Urethrostomy in Reconstructive Urology

**DOI:** 10.3390/jcm15114133

**Published:** 2026-05-27

**Authors:** Laura Velarde Ramos, Francisco Martins

**Affiliations:** 1 Department of Urology, Hospital del Trabajador, Ramón Carnicer 185, Santiago 7501241, Chile; 2 Department of Urology, Hospital Félix Bulnes, Avenida Mapocho 7432, Santiago 9081273, Chile; 3 Department of Urology, University of Lisbon School of Medicine, 1649-035 Lisbon, Portugal; faemartins@gmail.com

**Keywords:** perineal urethrostomy, urethral stricture, urethroplasty, urinary diversion

## Abstract

Perineal urethrostomy (PU) involves the creation of a urethral meatus at the perineal level. Traditionally, it was regarded as a last-resort therapeutic option or the outcome of failed previous reconstructive attempts. Currently, this perspective has evolved, and PU is increasingly considered a primary indication in complex cases or in patients with significant comorbidities. PU can be performed either as a definitive procedure—in patients with extensive, recurrent strictures or in those with comorbidities that preclude formal reconstruction—or as a temporary measure to facilitate healing and optimize conditions prior to definitive reconstructive surgery. In oncologic cases associated with total penectomy, PU serves as the definitive form of urinary diversion, and the same reconstructive principles should be applied to its design. A key requirement for PU is preserved sphincteric continence and the absence of proximal urethral stenosis. Advances in surgical techniques, including the use of flap mobilization and grafting, have enabled the creation of wide, functional meatuses, reducing the rate of stricture recurrence. Additionally, non-transecting variants aim to preserve the vascularization of the dorsal urethral surface. Following these surgical principles, high success rates can be achieved. Despite representing a significant alteration of anatomy and voiding habits, patient satisfaction is generally high. PU should not be viewed merely as a palliative alternative but rather as a versatile reconstructive option capable of providing comfortable and durable voiding in selected patients. Appropriate patient selection and meticulous surgical execution remain essential pillars of contemporary urethral surgery.

## 1. Introduction

Urethral stricture disease can lead to a significant deterioration in quality of life, associated with poor voiding, recurrent urinary tract infections, and impaired bladder function if left untreated.

PU is a type of urinary diversion that involves opening the urethra proximal to the stricture and creating a stoma in the perineum through which voiding occurs. This option is considered when the posterior urethra and the sphincteric complex remain intact [1,2].

Traditionally, PU was indicated in patients for whom other reconstructive urethral procedures were no longer feasible, particularly in elderly individuals and/or those with comorbidities that increased anesthetic risk, as well as in oncologic cases requiring penile amputation [3,4]. However, in recent decades its use has expanded in the management of complex urethral strictures, and it has even been considered as a primary, valid, and equivalent option within the reconstructive spectrum, rather than solely as a salvage procedure after failed urethral reconstruction [5,6,7].

Several surgical techniques have been described, with long-term success rates ranging between 70% and 90%. PU may be performed as either a temporary or definitive diversion, depending on the underlying etiology [8].

The advantages of PU, compared to other urethroplasty modalities, include reduced surgical complexity and operative time, faster postoperative recovery, and preservation of urinary continence. Its main drawbacks are the anatomical changes that necessitate voiding and ejaculation through the perineum [9].

Overall, the complication rate of PU is relatively low, with stomal stenosis being the most significant and challenging adverse event, often requiring surgical revision. Importantly, the anatomical alteration of voiding and ejaculation does not usually have a major emotional impact on patients, as the resolution of obstructive voiding symptoms—and the consequent improvement in quality of life remains their primary concern [2,8,9].

## 2. Indications

### 2.1. Non-Oncologic

The surgical management of complex urethral stricture represents a challenge, as not all patients are suitable candidates for reconstructive surgery due to the extensive length of the stricture, multiple recurrences, or associated comorbidities. In this context, definitive perineal urethrostomy (PU) constitutes a valid option for urinary diversion.

#### 2.1.1. Urethral Stricture

- Panurethral stricture: in patients with extensive urethral involvement due to lichen sclerosus or inflammatory/infectious etiologies, severe compromise of the urethral mucosa and corpus spongiosum may develop, often requiring staged reconstruction. These patients typically have undergone multiple prior treatments, such as repeated dilatations or endoscopic urethrotomies. In this setting, and after shared decision-making with the patient, PU represents an excellent alternative, even as a first-line option, since it provides a continent, non-obstructive urinary diversion in a single procedure [10,11,12].
- Recurrent stricture after multiple urethroplasty attempts: when no suitable tissue remains for adequate urethral reconstruction—after prior use of buccal mucosa grafts (cheek, lower lip, tongue), preputial grafts, and/or skin flaps—PU is an effective alternative. This group also includes patients with hypospadias who have undergone multiple surgeries without achieving satisfactory voiding, whether due to recurrence or persistent fistulous tracts. Some of these patients seek a definitive functional solution, prioritizing effective voiding through the perineal meatus over attempting to void through a diseased urethra [13].

#### 2.1.2. Comorbidities Contraindicating Extensive Urethral Reconstruction

Elderly patients with limited life expectancy and/or significant comorbidities, as well as those classified as ASA III by the American Society of Anesthesiologists (severe systemic disease with marked functional limitation), are at increased anesthetic risk. In such cases, a single, shorter procedure compared with urethral reconstruction is an appropriate option [14,15].

#### 2.1.3. Obesity and Buried Penis

Obesity poses an increased surgical risk and is sometimes associated with a buried penis, making voiding difficult and predisposing to urinary tract infections and dermatologic complications secondary to urine retention in the preputial sac after voiding [16]. In such cases, even in the absence of urethral stricture, PU may be considered as a urinary diversion to avoid catheter use.

#### 2.1.4. Traumatic Penile Amputation

In cases of traumatic penile amputation, whether accidental or self-inflicted, when the residual stump is very proximal and does not allow adequate projection of the urinary stream resulting in a dispersed rather than forward directed stream PU is recommended [17].

#### 2.1.5. Others

Certain neurologic diseases or sequelae may limit the ability to void in the standing position, such as advanced Parkinson’s disease or hemiplegia or some genetic conditions associated with severe disabilities, where caregivers report that patients habitually void in the sitting position. In such cases, if a urethral stricture of any etiology develops, PU may be considered a treatment option.

### 2.2. Oncologic

#### 2.2.1. Penile Cancer: Its Incidence Varies Worldwide

It is uncommon in industrialized countries but higher in parts of South America, Southeast Asia, and some regions of Africa. Surgical treatment involves excision of the primary lesion and, in some cases, total penectomy, which requires urinary diversion with PU [18]. After partial penectomy, a small penile stump may remain that is difficult to direct the urinary stream forward; in such cases a PU may be a reasonable option.

#### 2.2.2. Urethral Cancer

Its incidence accounts for <1% of all genitourinary malignancies in men, peaking in patients older than 75 years. Partial or total penectomy was once the standard of care, but more conservative, phallus-preserving techniques have demonstrated good oncologic control [19]. However, when urethrectomy must be performed proximally, with or without phallic preservation, the neomeatus may be positioned unfavorably for directing the urinary stream; in these cases, after discussion and agreement with the patient, a PU may be considered.

#### 2.2.3. Bladder Cancer

Patients with non–muscle-invasive bladder cancer require protocolized regular cystoscopic surveillance, may require intravesical instillations, and if recurrence occurs, repeat endoscopic resection. If urethral stricture coexists, the need for urethral reconstruction should be evaluated either at this stage or deferred until oncologic control is achieved. If urethroplasty has been performed in this context, urethral instrumentation is undesirable; a PU is a good option for urinary diversion during this period of treatment and follow-up, avoiding manipulation of the diseased urethra and dilations that may increase stricture length and/or spongiofibrosis, complicating subsequent reconstruction [20,21].

## 3. Types

The first description in the literature of a perineal urostomy (PU) was made in 1914 by Russell [22], who described the concept of urethral marsupialization as a definitive management for stricture. Subsequently, this concept was used as the first part of a staged urethroplasty by Johanson in 1953 and Turner-Warwick in 1960 [23,24]. Over time, different techniques were developed to create a perineal meatus, either by transecting or not transecting the urethra.

### 3.1. Transecting or Non-Transecting PU

**Figure 1:**
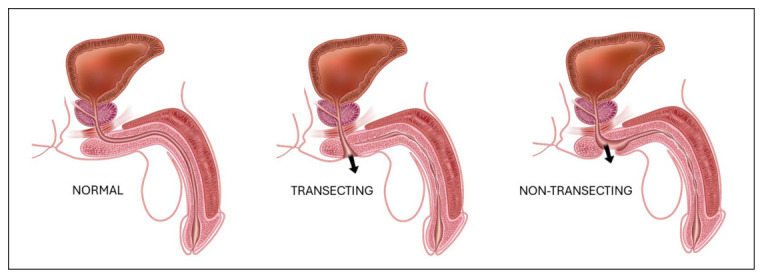
Urethral transection.

Classic techniques are described with complete transection of the urethra. The concept of non-transection involves opening the urethra on the ventral surface while preserving the dorsal surface and, consequently, its vascularization [25]. This also allows for the creation of wide meatuses. The first technique described was urethral marsupialization through a longitudinal incision, which was introduced by Johanson as the first step in a staged reconstruction [23]. Its main difficulty lies in cases where the stricture is very proximal, as the stoma can be under tension. Subsequently, modifications of various techniques were carried out, allowing some of them to be performed without urethral transection while maintaining the fundamental concepts of the original surgery (Figure 1).

### 3.2. Techniques Using Cutaneous Flaps

- Blandy [26]: This technique was originally described as transecting, characterized by the creation of a perineal meatus through mobilization of a cutaneous flap in an inverted “U” shape. Modifications were then made based on the original technique, including the lambda incision and non-transection of the urethra.
- “7” Flap [27]: This technique was described in 2011 by French et al. It describes the creation of a perineal meatus by mobilizing a cutaneous flap shaped like a “7.” This technique is particularly useful in obese patients and in those cases indicated for urethral reconstruction via a median perineal incision. However, during surgery, it was determined not to progress with the reconstruction and to perform a PU instead. This technique allows for the widening of the incision in the shape of a “7” and the mobilization of the cutaneous flap to achieve a wide, tension-free meatus. The reported success rate of this technique is greater than 95%.
- Lotus Petal Flap [28]: This technique was initially described for the reconstruction of vulvovaginal defects, with very good results. This concept was extrapolated to the creation of a male perineal meatus in complex cases. It was originally described by French and later modified by Reilly et al. in 2018, using mobilization of regional myocutaneous or adipose flaps with a perforator-based blood supply from its base. It is indicated in complex cases with scarred perineum, stenosis of the perineal meatus, and in obese patients. Surgically, this technique is more challenging than the others as it requires experience in identifying the vascular pedicle of the flap, but it allows for the flap to be brought to the urethrotomy and creates a wide meatus with minimal tension. A disadvantage of this technique is the potential presence of hairs in the meatal pathway.
- Perineal Z-plasty [29]: This non-transecting technique involves creating a median perineal incision, with incisions on both sides at a 45° angle, creating a “Z” shape that allows for the mobilization and rotation of the flaps, achieving a tension-free stoma.

### 3.3. Techniques Using Grafts

- Oral Mucosa Graft [30,31]: The use of oral mucosal grafting in perineal urethrostomy was described in 2008 by Kamat and modified by DeLong et al. in 2017. Unlike techniques involving the mobilization of cutaneous flaps towards the urethra, in this technique the urethrotomy is superficialized and complemented with a graft to create a wide meatus. The use of an oral mucosa graft is highly versatile, as it can be applied in transecting techniques by placing the graft distally, or in non-transecting techniques, where grafts are used to enlarge the meatus along its lateral aspects and/or its superior or inferior surface. The reported success rate of this technique is over 80%.
- Mesh Skin Graft [32]: Described by Lumen in 2014 for patients in whom a PU is considered, but the skin of the perineum is pathological or scarred. This skin is not suitable for tissue mobilization; hence, it is proposed for removal and replacement with a graft of healthy skin that will surround the perineal meatus.

## 4. Preoperative Preparation

At this stage, in addition to assessing the patient’s general conditions for surgery, other needs must be considered, not only to reduce risks and prevent complications arising from the procedure but also to identify aspects related to self-care, mental health, and the patient’s support network.

### 4.1. Clinical Evaluation

This includes the development of detailed medical history, a complete physical examination, and an assessment of the general health status. Chronic diseases such as diabetes mellitus, hypertension, and heart disease must be well-controlled.

### 4.2. Additional Diagnostic Studies

Laboratory blood tests, urinalysis, urine culture, and imaging studies such as retrograde urethrocystography.

### 4.3. Medical-Patient Counseling

This is an important aspect, as it is essential to explore the patient’s expectations and priorities. The primary objective of the surgery should be adequately communicated, and the anatomical changes that the procedure will entail concerning urination and ejaculation should be explained in detail. It is also advisable to address sexual aspects, as a common concern is the possibility of erectile dysfunction following surgery [33,34].

### 4.4. Psychological Support

Undergoing this procedure involves a significant anatomical change that not only affects the ability to urinate while standing but also impacts ejaculatory function. In oncological patients who may also require penile amputation, psychological support becomes crucial [35].

### 4.5. Informed Consent

Once the clinical evaluation, medical-patient counseling, and assessment of the need for psychological support are completed, the informed consent explanation should be conducted. This must clearly describe the purpose of the procedure, the risks, benefits, and alternatives, and must be signed by both the patient and the treating physician.

## 5. Surgical Technique

### 5.1. Anatomical Landmarks

**Figure 2:**
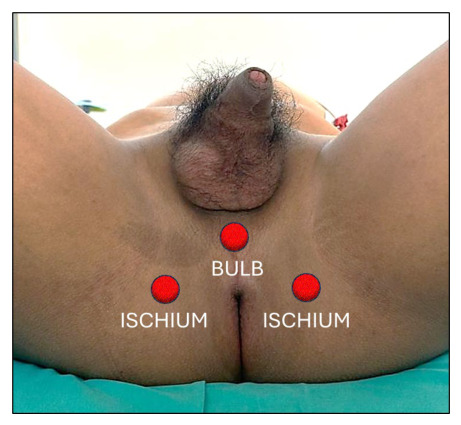
Anatomical landmarks.

The design of the perineal urethrostomy (PU) is the cornerstone for ensuring the correct location of the neomeatus. The main reference points are the ischial tuberosities and the urethral bulb (Figure 2). An adequate design allows the urinary stream to be directed downward with minimal dispersion. However, if the meatus is located in the distal urethra, it may end up within the scrotum, in thicker skin, directing the stream forward. This results in a non-functional outcome for the patient, since even when voiding in a seated position, the stream projects forward.

### 5.2. Description of Techniques

- Johanson technique [23]: the least complex technique, consisting of urethral marsupialization without the need for cutaneous flap mobilization, unlike the other approaches. It involves a midline perineal incision, division of the bulbospongiosus muscle, proximal bulbar ventral urethrotomy, watertight closure of the corpus spongiosum, and urethral mucosa anastomosis to the perineal skin to create the neomeatus.
- Blandy technique [26]: characterized by the creation of the meatus through mobilization of a posterior perineal flap in an inverted “U” shape, whose vascularization depends on the superficial perineal artery. These principles may be applied either with or without urethral transection.
- “7”-shaped flap [27,36]: This procedure is performed through a midline perineal incision extended superiorly with a “7”-shaped marking. After exposure of the urethra, it may be transected or left in continuity. A laterally based full-thickness skin flap is created according to the “7”-shaped design and advanced toward the lateral aspect of the urethrotomy. The remaining skin is used to complete the mucocutaneous anastomosis.
- Lotus petal flap [28]: It involves urethral transection with the aim of bringing the proximal urethral end closer to the perineum through flap interposition. To this end, perforating vessels from the internal pudendal artery are identified using Doppler ultrasonography. The urethra is debrided until healthy tissue is reached at the proximal end, which is then spatulated. The distance from the perineal skin to the urethra is measured, and a lotus petal–shaped flap is designed incorporating the previously identified perforators. The flap is mobilized in a suprafascial plane from distal to proximal, preserving the cutaneous bridge at its base. It is then inset in a semicircumferential or fully circumferential fashion between the urethral edge and the perineal skin, and the donor site is closed.
- Perineal Z-plasty [29]: used to relieve tension on perimeatal tissues. Two angled incisions are made over the midline perineal incision, forming a “Z” and creating two flaps, labeled A and B, which are rotated in opposite directions: flap A downward and flap B upward.
- Augmented PU with oral mucosa graft [31]: The perimeatal graft creates a continuity effect with the urethral mucosa, which is particularly useful in cases where the proximal end cannot be spatulated, resulting in a small meatus. The dorsal aspect of the urethra is extended with a graft anchored to the corpora cavernosa and sutured to the skin, giving the meatus a teardrop-shaped configuration. In some cases, it is necessary to complement this with additional grafts on the lateral aspects of the meatus, or even circumferentially, in order to achieve a wide meatus.
- Augmented PU with meshed skin graft [32]: In this technique, pathological perineal skin is replaced with healthy skin, creating a meatus whose edges are sutured to the graft. A split-thickness skin graft is harvested using a dermatome, placed on a surface, and meshed using a dedicated device to produce microincisions that allow stretching of the graft and coverage of a larger area. A meshed skin graft facilitates adaptation and may accelerate the healing process compared with a non-meshed graft. One characteristic of these grafts is that they leave a finely textured surface at the graft site; however, in the context of a PU, this feature does not represent a disadvantage.

After the PU procedure, a urethral catheter is left in place for 5 days to 2 weeks [23,26,27,28,29,31,32].

**Figure 3:**
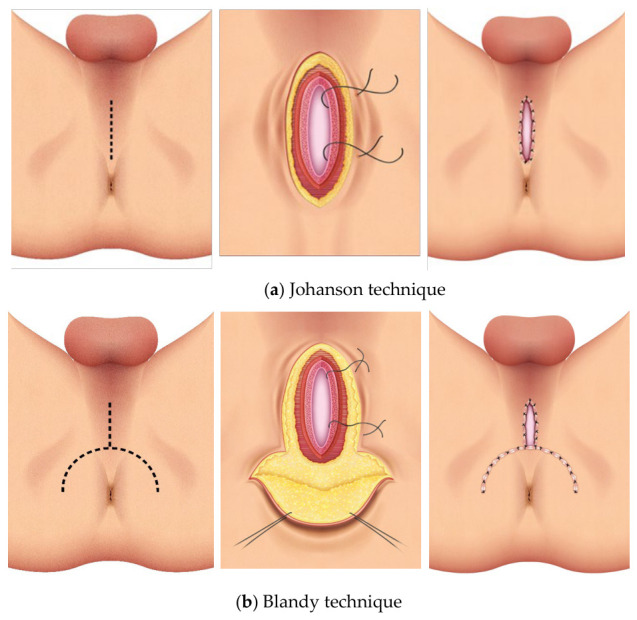

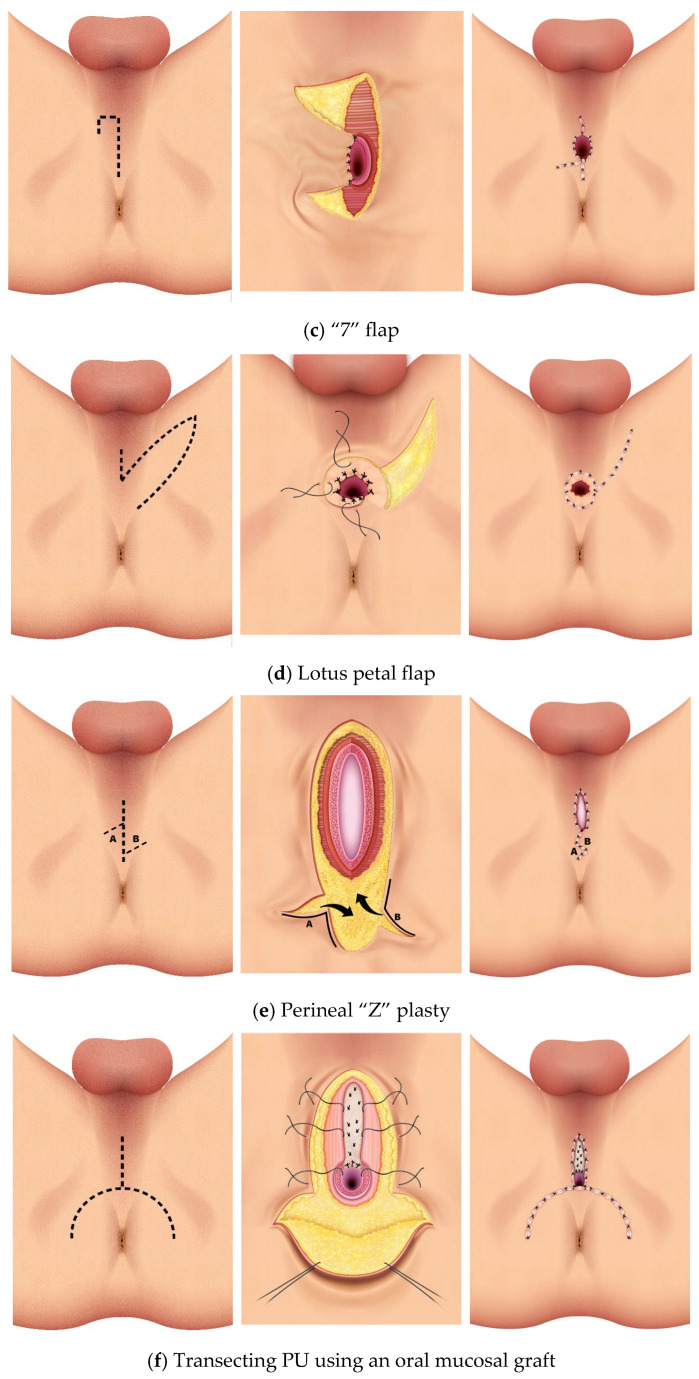

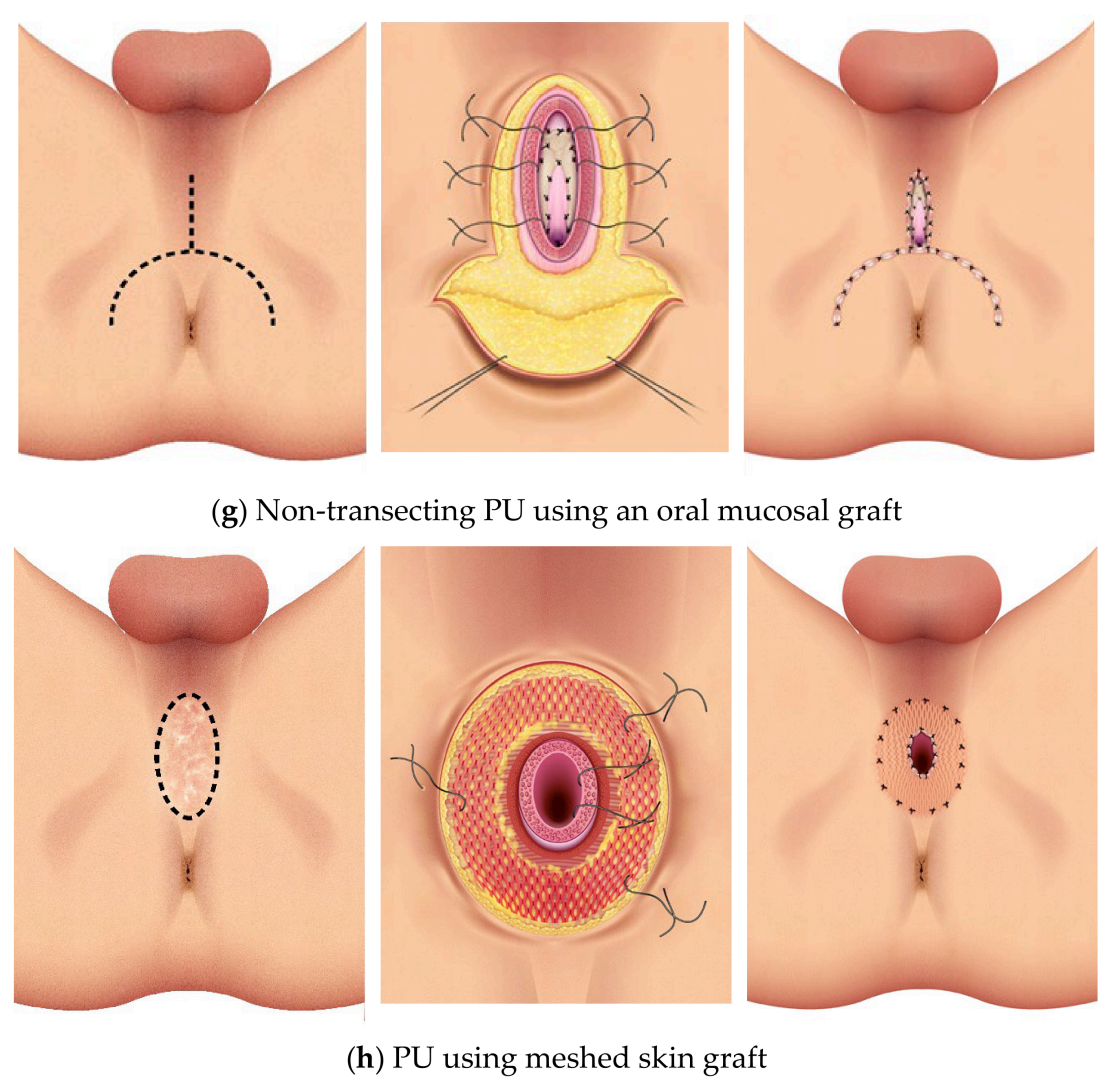
Schematical representation of surgical techniques.

Figure 3 schematically illustrates the different surgical techniques, and Table 1 describes their advantages and disadvantages.

## 6. Complications

PU has a low overall incidence of complications [8,9,37,38].

Early complications include bleeding due to inadequate spongiosal closure, hematoma, superficial wound dehiscence, scrotal edema, and perineal pain, most of which are classified as Clavien-Dindo grade I or II [8,33]. Clavien-Dindo grade III complications have been reported in approximately 6% of cases [39].

Among late complications, the most relevant is neomeatal stenosis, with an incidence of up to 30% [8,11,25]. Risk factors include prior infectious etiology [8], perineal trauma, radiotherapy—associated with up to a 12-fold higher risk of stenosis [25]—and history of Fournier’s gangrene involving the perineum.

Regarding lichen sclerosus, some authors report PU success rates between 93% and 100% [7,12,34]. Others, however, consider it a risk factor for perineal meatal stenosis [8], with a threefold higher likelihood of requiring repeat surgery compared with patients undergoing PU for idiopathic strictures or strictures secondary to hypospadias repair [15], particularly when lichen extends to the bulbar urethra.

PU effectiveness is also reduced in patients with recurrent strictures following multiple failed endoscopic or reconstructive surgeries [40,41,42].

Concomitant chronic diseases may compromise surgical outcomes: ischemic heart disease doubles the risk of stenosis [15], while arterial hypertension has also been associated with a higher incidence of complications.

Age-related outcomes are controversial. Some authors suggest that older patients develop fewer strictures [8], whereas others consider advanced age a negative prognostic factor [14], reporting that no patients under 50 years developed stenosis, in contrast with 83% of patients aged 60 to 70 years.

Patient satisfaction: although the anatomic modification of voiding and ejaculating through the perineum may initially have significant emotional impact, the literature demonstrates that, in the long term, patient satisfaction with surgical outcomes and quality of life is high. Across all reviewed studies, patients reported no regret regarding surgery and indicated they would recommend it to others in a similar situation [8,9,10,15].

Correction of perineal meatal stenosis primarily depends on the quality of perineal skin, which guides the choice of technique. Options range from reconstruction of the meatus using classical Blandy flaps to the design of alternative flaps that relieve anastomotic tension. In other cases, enlargement of the stoma or substitution of diseased tissue with grafts may be required [43,44,45].

## 7. Discussion

Traditionally, PU was considered the last resort in the management of urethral stricture disease. However, several studies have demonstrated that, in recent decades, its use as a definitive treatment has increased, particularly in complex cases. This trend is largely due to the fact that many patients are unwilling to undergo staged urethral reconstruction, especially those with prior failed surgeries, whose primary goal is to achieve unobstructed urinary flow with a single procedure [5].

It has been reported that nearly 50% of patients initially indicated for staged urethral reconstruction decide not to proceed with subsequent stages, as they are satisfied with voiding outcomes following PU. Age also plays a role, as younger patients without comorbidities more frequently opt for complex reconstructive procedures [8,10].

This scenario has shifted the approach to urethral reconstruction, emphasizing not only surgical aspects but also patient expectations and quality-of-life considerations. Therefore, during the initial evaluation, it is essential to explore the patient’s expectations regarding the reconstructive process, anticipated outcomes, and potential complications.

Proper patient selection for different reconstructive techniques is key to achieving favorable outcomes. Long-term results of PU are well documented, showing success rates exceeding 80% with satisfactory quality of life [1,2,7,8,9].

Regarding the choice of PU technique, no significant advantages have been documented among the various approaches. The indication depends mainly on whether the bulbar urethra can be easily exposed in the perineum to create a neomeatus, or whether additional flaps or grafts are required to extend the tract.

In certain urethroplasties, intraoperative challenges may necessitate performing a PU. In cases where a midline perineal incision is made and tissue mobilization is required to achieve a tension-free neomeatus, several flap designs have been described, all demonstrating favorable outcomes [29].

Complications of PU, such as wound dehiscence or infection, are less frequently observed with a midline incision compared to the inverted “U” incision [44].

The current European Association of Urology (EAU) guidelines recommend PU as an independent treatment option for men with complex anterior urethral strictures, as well as for those unwilling to undergo staged reconstruction or with comorbidities contraindicating lengthy procedures. Furthermore, the guidelines emphasize that the choice of technique depends on surgeon experience and stricture characteristics. For proximal strictures, augmentation PU using Blandy’s technique or a “7-flap” design is recommended, whereas in obese patients, the “7-flap” is preferred [1].

The American Urological Association (AUA) guidelines provide similar recommendations, endorsing PU in cases of primary or recurrent complex strictures, advanced age, significant comorbidities precluding prolonged operative times, extensive lichen sclerosus, multiple failed urethroplasties, and/or strong patient preference [46,47].

## 8. Conclusions

PU represents an effective and definitive therapeutic option in cases of extensive and recurrent urethral stricture, as well as in patients with significant comorbidities that increase surgical risk. It is also indicated in the oncologic setting when penile amputation is required. Furthermore, it constitutes an excellent temporary alternative in patients with bladder cancer who will require multiple urethral instrumentations.

Functional outcomes are well documented, with an acceptable complication profile. Most patients achieve satisfactory voiding, with minimal need for additional interventions.

Standardization of the technique and surgeon experience are key factors in optimizing outcomes. In this regard, particular attention should be paid to the creation of a tension-free perineal meatus through adequate mucocutaneous anastomosis.

## Figures and Tables

**Table 1:** Advantages and disadvantages of the surgical techniques for PU.

	Advantages	Disadvantages
**Johanson**	Easy technique, without flap mobilization or graft use.	PU stenosis
**Blandy**	High success rate	More complex technique due to flap mobilization
**“7” Flap**	Obese patients Conversion of a midline perineal incision during an intraoperative change in surgical plan	More complex technique due to flap mobilization
**Lotus Petal Flap**	Connection of the urethral meatus to the perineal skin	Complex technique Identification of flap vascularization using Doppler ultrasound.
**“Z” Plasty**	Tension-free wound	
**Oral Mucosal Graft**	Continuity of the urethral mucosa with oral mucosa Wide meatus.	Possible graft retraction
**Meshed Skin Graft**	Replacement of diseased skin with healthy skin. Wide coverage using a meshed graft.	Complex technique including skin graft harvesting. Grafted skin with a granular texture.

## Data Availability

No new data were generated or analyzed in support of this research.
